# Screening and characterisation of sex differentiation-related long non-coding RNAs in Chinese soft-shell turtle (*Pelodiscus sinensis*)

**DOI:** 10.1038/s41598-018-26841-3

**Published:** 2018-06-05

**Authors:** Jun Zhang, Peng Yu, Qinyan Zhou, Xilei Li, Shuquan Ding, Shiping Su, Xiaohua Zhang, Xiaoli Yang, Weishang Zhou, Quan Wan, Jian-Fang Gui

**Affiliations:** 1 College of Animal Science and Technology, Anhui Agricultural University, Hefei, 230036 China; 2 State Key laboratory of Freshwater Ecology and Biotechnology, Institute of Hydrobiology, Chinese Academy of Sciences, Wuhan, 430072 China; 3 University of Chinese Academy of Sciences, Beijing, 100049 China

## Abstract

Long non-coding RNAs (lncRNAs) perform distinct functions in various biological processes in mammals, including sex differentiation. However, the roles of lncRNAs in other vertebrates, especially in the Chinese soft-shell turtle (*Pelodiscus sinensis*), remain to be clarified. In this study, we performed genome-wide analysis of the lncRNA expression profiles in gonad tissues and screened numerous sex-specific lncRNAs in the Chinese soft-shell turtle. Of the 363,310,650 clean reads obtained, 5,994 sequences were typed as lncRNAs, of which 4,463 were novel. A selection of sex-specific lncRNAs (♀ 932, ♂ 449) from female ovaries and male testis were shown to act on target genes in *cis* and in *trans*, and most were involved in gonad differentiation based on Kyoto Encyclopedia of Genes and Genomes (KEGG) analysis. Furthermore, interactions among the differentially expressed lncRNA-mRNAs and protein coding genes were identified by construction of correlation networks. Overall, our systematic analysis of lncRNA expression profiles in gonad tissues revealed numerous sex-specific lncRNAs in *P*. *sinensis*. Thereby, these findings provide new insights into the function of lncRNAs in sex differentiation and highlight a group of candidate lncRNAs for future studies.

## Introduction

The mechanisms underlying sex determination are an important focus of biological research^1–3^. Most vertebrates are gonochoristic, with sexual morphology determined genetically by different alleles or genes^4^. In single gene systems, sex chromosomes contribute to sex determination, with the XX/XY male and ZZ/ZW female heterogametic systems present in the majority of species, although variant systems have been identified in a few species^2^. Sex determination, which results in the formation of testes and ovaries from undifferentiated gonads, is generally thought to be genetically controlled but can also be influenced by environmental factors (such as temperature) or social variables (e.g. the relative size of an organism in its population)^5–7^. The sex-determining (SD) genes that control this process are expressed transiently in undifferentiated gonads. The first SD to be discovered in vertebrates was the *Sry* gene, which is located on the Y-chromosome and initiates testicular differentiation^8^. Other SD genes or candidates include *dmy*^9,10^, *amhr2*^11^, *amhy*^12^, *gsdf*^13,14^, *sox3*^13^ and *dmrt1*^15,16^.

LncRNAs have been implicated in many biological processes and function through the regulation of transcriptional and post-transcriptional gene expression^17–20^. Accumulating evidence suggests that lncRNAs play a role in sex determination and gonadogenesis^21,22^, meiosis^23^, gametogenesis^21,24,25^, cell differentiation and organogenesis^26^ and sexual reproduction^18,27^. Of note, *Xist* lncRNAs mediates transcriptional silencing of one X-chromosome during female sex determination in mammals^28^. In mice, the lncRNA *Dmr* has been shown to participate in *trans* splicing of the *Dmrt1* transcript that encodes a protein with an altered carboxyl terminus^29^. Furthermore, a complex network of numerous lncRNAs regulate *Sxl* expression^30^. All of these observations indicate that lncRNAs play important roles in sex differentiation.

Reptiles exhibit an extraordinary variation in the mechanisms of sex determination including temperature-dependent and genetic sex determination (TSD and GSD, respectively) mediated by the male (XY) or female (ZW) heterogametic systems^31,32^. However, the potential roles of lncRNAs in sex differentiation of reptiles remain to be elucidated. The Chinese soft-shell turtle (*P*. *sinensis*) is a member of the turtle family (Reptilia; Testudines) and is an important aquaculture species in southern China. This species has heteromorphic ZW-type micro-sex chromosomes^31,33,34^ and, as with most vertebrates, sex determination controls the development of testes or ovaries from the bipotential gonad^35^. Sex differentiation-related genes, such as *Dmrt1*, *Amh*, *Sox9*, *Cyp19a1* and *Foxl*2 were discovered in Chinese soft-shell turtle. Furthermore, *Dmrt1* appeared early male gonads specific expression, which was knockdown in ZZ embryos by RNA interference resulted in male to female sex reversal^33^. Although a series of sex-differentiation-related genes were discovered in Chinese soft-shell turtle, the potential involvement of lncRNAs in sex determination of this species remains to be clarified.

Chinese soft-shell turtles have a sex-dependent dimorphic growth pattern, with males exhibiting more rapid growth and larger body size, with a thicker and wider calipash, and lower levels of fat^33^. This pattern of sex-related differences in growth rate is also observed in many species of farmed teleost fish, such as yellow catfish, and strategies for sex control have been used to produce all-male populations^36,37^. Similar approaches have been investigated in turtles. Therefore, a comprehensive understanding of the mechanisms responsible for sex determination in Chinese soft-shell turtles will be of significant benefit for improved aquaculture of this species. Here, we systematically analysed the lncRNA expression profiles in gonad tissues and screened numerous sex-specific lncRNAs in Chinese soft-shell turtles. This information may provide a better understanding of the sex differentiation processes in Chinese soft-shell turtle and also highlight the functions of lncRNAs in this process in all reptiles.

## Results

### Overview of lncRNA sequencing

For genome-wide analysis of lncRNAs of *P*. *sinensis*, gonad tissues collected at 30 days post-hatching were subjected to RNA-seq using the Illumina HiSeq. 4000 platform. In total, 363,310,650 clean reads were produced and 354,585,240 high quality (HQ) clean reads were obtained by removing low quality reads and reads mapped with rRNA. Between 63.66% and 67.98% of the HQ reads were efficiently mapped against the *P*. *sinensis* reference genome (PelSin_1.0, NCBI) in each library, thus validating our RNA-sequencing data (see Supplementary File S1).

### Identification of lncRNAs and mRNAs

**Figure 1:**
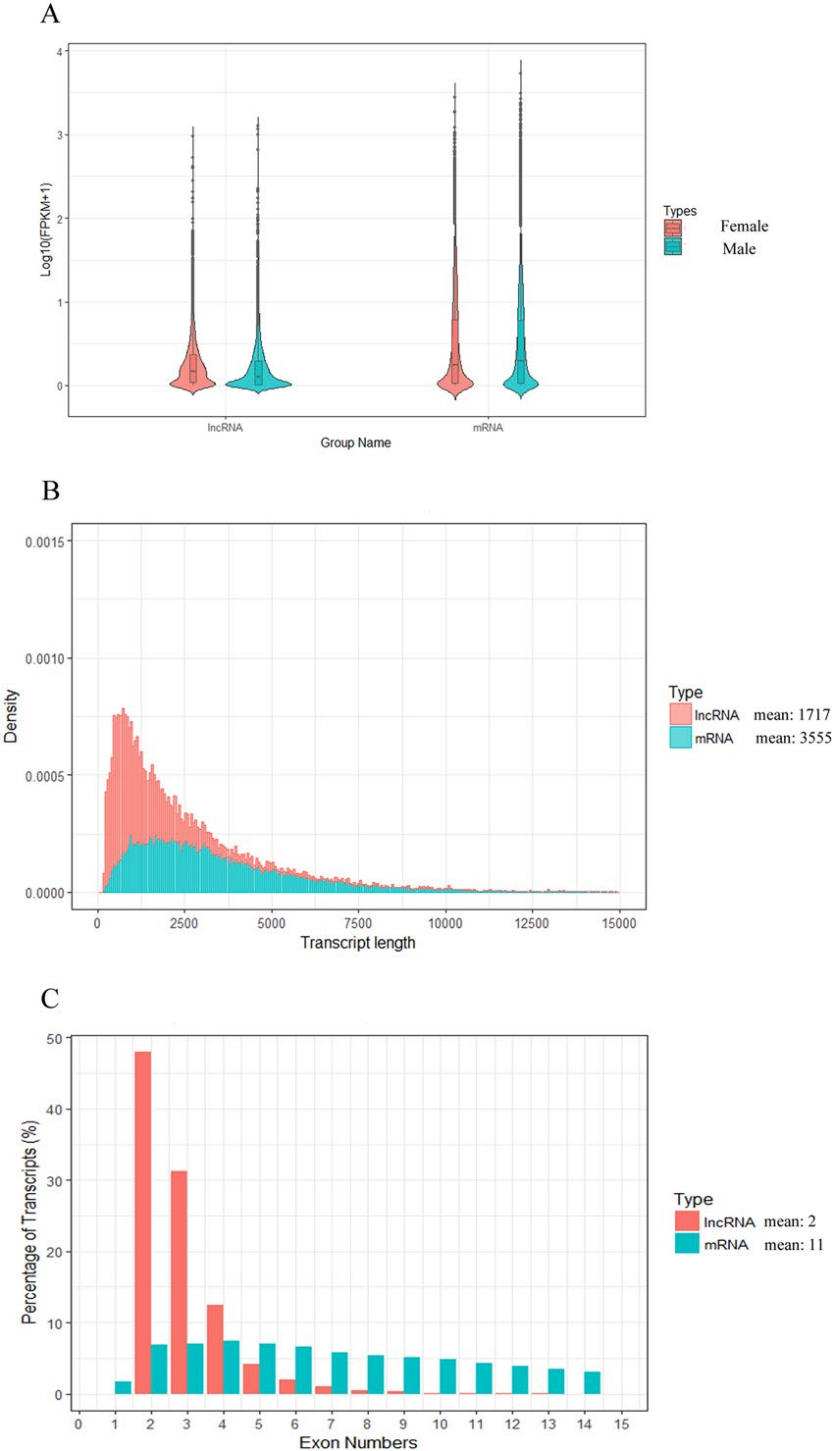
Comparison of features of predicted lncRNAs and mRNAs. (**A**) Expression of lncRNAs and mRNAs. (**B**) Length distribution of predicted lncRNAs and mRNAs. (**C**) Exon number distribution of lncRNAs and mRNAs.

A high stringency filtering process was used to remove low quality lncRNA transcripts (see Supplementary Fig. S1). Of the remaining 5,994 lncRNA transcripts, only 1,531 (25.54%) were previously identified, while 4,463 (74.46%) were novel. In contrast, the Illumina RNA-seq analysis produced a much higher number of mRNAs (39,262), comprising 27,429 known transcripts and 11,833 novel transcripts (Fig. 1A). The mean length of lncRNAs in our dataset was 1,717 bp and the mean mRNA length was 3,555 bp (Fig. 1B). Furthermore, the lncRNAs contained fewer exons than the mRNAs (Fig. 1C).

### Sex-specific expression of lncRNAs and mRNAs

**Figure 2:**
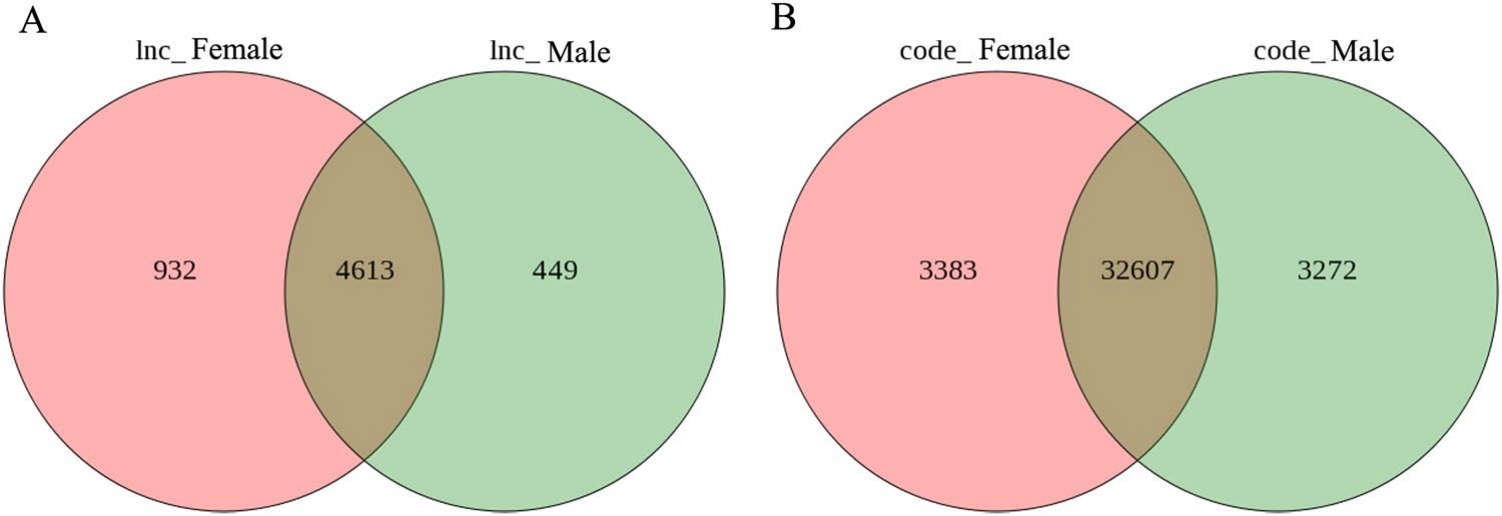
Analyses of differentially expressed lncRNAs and mRNAs. (**A**) Venn diagram showing the differentially expressed lncRNAs in female and male gonad tissues. (**B**) Venn diagram showing the differentially expressed coding transcripts in female and male gonads.

Analysis of sex-specific gene expression revealed 932 lncRNAs expressed specifically in the female gonad tissues and 449 in the male samples (Fig. 2A). We also identified 3,383 mRNAs expressed specifically in female gonad tissues and 3,272 in male gonad tissues (Fig. 2B). These lncRNAs and mRNAs may play a pivotal role in *P*. *sinensis* gonad formation and provide crucial information regarding the regulation of sex differentiation in this species.

### Analysis of differentially expressed lncRNAs and mRNAs

**Figure 3:**
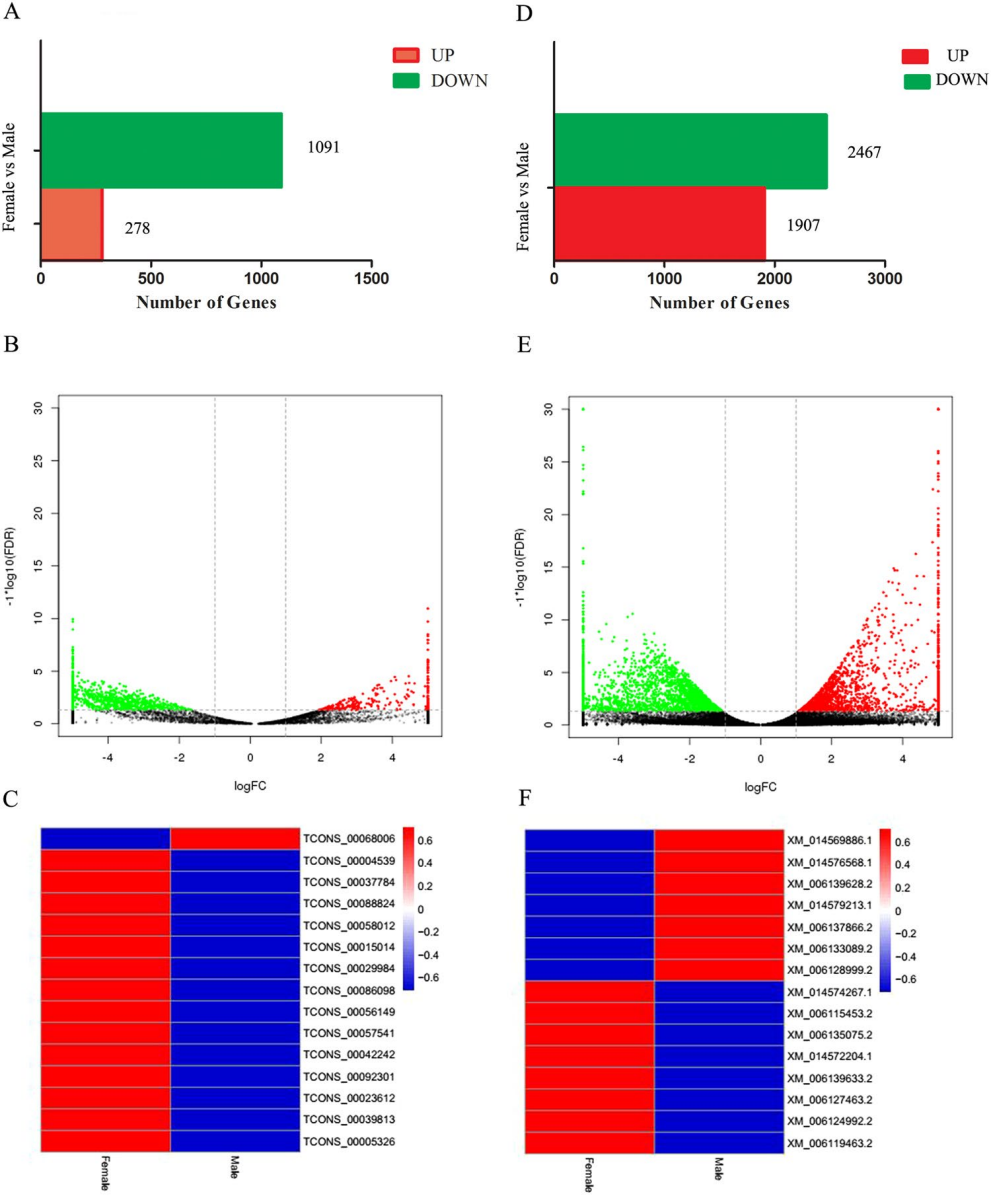
Differentially expressed transcripts in the RNA-seq libraries. (**A**) and (**D**) Numbers of up-regulated (red) and down-regulated transcripts (green). (**B**) and (**E**) Volcano plots of differentially expressed transcripts. X-axis represents fold change (log 2) and Y-axis represents *P* (−log 10). Red points indicate up-regulated (X-axis >0) transcripts; green points indicate down-regulated (X-axis <0) transcripts. (**C**) and (**F**) Heatmaps showing 15 sex differentiation-related lncRNAs and mRNAs. The data are depicted as a matrix, in which each row represents one lncRNA (mRNA) and each column represents one group. The relative expression of lncRNA (mRNA) expression is depicted according the colour scale shown on the right. Red represents high relative expression, and blue represents low relative expression; −0.6, −0.4, −0.2, 0 and 0.2, 0.4, and 0.6 are fold changes in the corresponding spectrum. The magnitude of deviation from the median is represented by the colour saturation.

The RNA-seq data were analysed to further clarify the differential expression of transcripts in female and male gonad tissues. A total of 1,369 lncRNA transcripts were differentially expressed in male tissues compared with the levels detected in female tissues. Of these, 278 were up-regulated and 1,091 were down-regulated (fold change ≥2.0 and FDR <0.05; Fig. 3A). Volcano plots also revealed a similar trend (Fig. 3B). Of these, the 15 sex differentiation-related lncRNAs showing the most significant differential expression were selected for construction of a heatmap (Fig. 3C). Among the dysregulated sex differentiation-related lncRNAs, *TCONS_0009*2301 was the most down-regulated (log2(FC) -5.73649) and *TCONS_00068006* was the most up-regulated (log2(FC) 3.760947) (details are presented in Supplementary File S2 and S3).

In further analysis, we identified 4,374 mRNAs showing significant differential expression in male tissues compared with the levels detected in female tissues (1,907 up-regulated and 2,467 down-regulated (Fig. 3D and Supplementary File S2). A similar differential expression trend of mRNAs was observed in the volcano plot (fold change ≥2.0, *FDR* <0.05) (Fig. 3E). Of these, the 15 sex differentiation-related mRNAs showing the most significant differential expression were selected for construction of a heatmap (Fig. 3F). Among the dysregulated sex differentiation-related mRNAs, *XM_0*1*4578590*.*1* was the most up-regulated (log2(FC) -9.5411) and *XM_006133089*.2 was the most down-regulated log2(FC) 9.41996) (see Supplementary File S4).

### Putative target genes of lncRNAs

**Figure 4:**
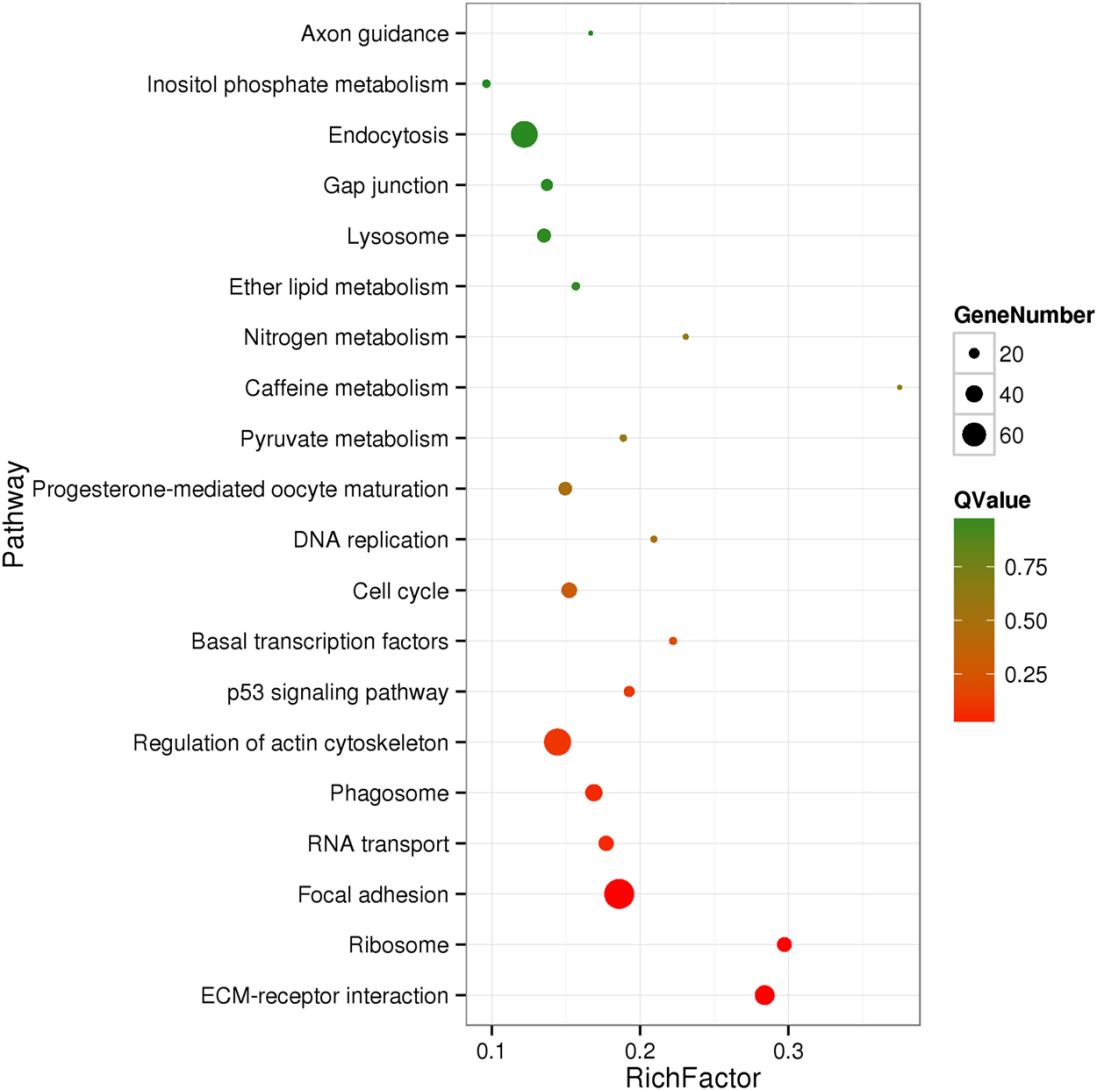
Top 20 KEGG pathway annotation categories for target gene functions of predicted lncRNAs.

LncRNAs regulate target gene expression by acting in *cis* on neighbouring loci or in *trans* on distant loci^38^. To investigate the effects of differences in lncRNAs on the functional regulation of turtle sex differentiation, we predicted the target genes of lncRNA using the *cis* and *trans* model. The *cis* role of lncRNAs was investigated by screening the protein coding (PC) genes as potential targets in the regions located 10-kb upstream and downstream of all the identified lncRNAs for prediction of their functional roles. In total, 933 lncRNAs were predicted to correspond with 1,179 target genes in *cis* and 5,990 lncRNAs were predicted to correspond with 6,609 target genes in *trans* (see Supplementary File S5 and S6). Interestingly, many lncRNAs were predicted to target sex differentiation-related genes; these included *TCONS_00099*273, *TCONS_00068006*, *TCONS_00088824*, *TCONS_000*19214, and *TCONS_0001944*2, which targeted the PC genes *dmrt*1, *sox9*, *cyp19a*, *sox3*, and *sox8*. These findings indicated that the on-set of sexual differentiation is regulated by the lncRNA-target genes. Regarding the *trans* role of lncRNA, our results indicated that the lncRNA, *TCONS_00071083*, acted on *ZFX* in *trans* (Table 1). Furthermore, KEGG pathway analysis of the top 20 enriched lncRNAs may be acting on target genes that regulate the onset of sex differentiation (Fig. 4 and Supplementary File S7).

**Table 1:** LncRNAs and their potential target genes associated with sex differentiation.

Target genes	*cis*	*trans*
*DMRT*1 *(XM_006*1*37866*.2*)*	*TCONS_00099*2*73*	
*CYP19a (XM_006135075*.*2)*	*TCONS_00088824*	
*GATA4 (XM_014569886*.*1)*		*TCONS_00057541* *TCONS_00039813* *TCONS_00056149* *TCONS_00005326* *TCONS_00037784* *TCONS_00015014* *TCONS_00023612* *TCONS_00004539* *TCONS_00092301* *TCONS_00086098* *TCONS_00029984* *TCONS_00058012*
*SOX3 (XM_006115453*.*2)*	*TCONS_00019214*	
*SOX8 (XM_006139628*.*2)*	*TCONS_00019442*	
*SOX9 (XM_014576568*.*1)*	*TCONS_00068006*	
*HOXD1 (XM_006119463*.*2)*	*TCONS_00032150*	
*HOXB13 (XM_014572204*.*1)*		*TCONS_00042242* *TCONS_00035891*
*ZFX (XM_006112508*.*2)*		*TCONS_00071083*
*SOX5 (XM_014572620*.*1)*	*TCONS_00039122* *TCONS_00039123*	
*RSPO1 (XM_006134251*.*2)*		*TCONS_00094168*

### Network of lncRNA and mRNA interactions

**Figure 5:**
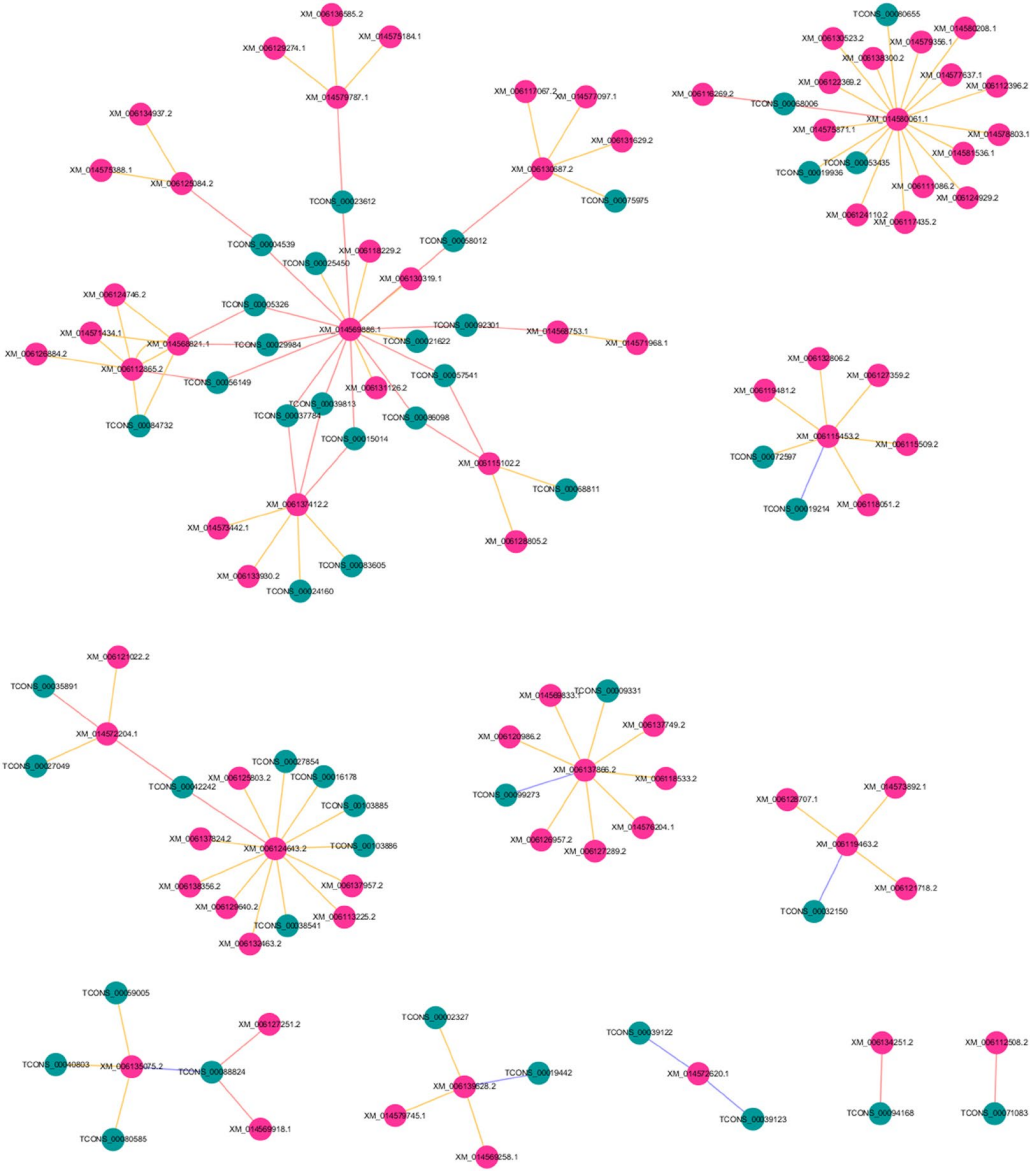
Correlation network of lncRNAs and mRNAs. Green and pink nodes represent dysregulated lncRNAs and dysregulated mRNAs, respectively. Blue and red lines between lncRNAs and mRNAs indicate *cis* and *trans* actions, respectively. Yellow lines represent Pearson correlation coefficients >0.99999.

To identity more important genes related to sex differentiation, we constructed a correlation network of the interactions between the differentially expressed lncRNAs and mRNAs (*P* > 0.99999 in Pearson correlation analysis) based on *cis* or in *trans* regulation (Fig. 5). The correlation network was constructed from 63 lncRNAs and 185 mRNAs associated with sex differentiation-related dysregulated genes. A review of the network showed that lncRNAs regulated target genes either in *cis* or in *trans*. For instance, the PC gene *XM_00611*2508.2 was targeted by lncRNA *TCONS_0007*1083 in *cis*, while the PC gene *XM_0*1*457*2*6*2*0*.1 was targeted by lncRNA *TCONS_000391*2*3* and *TCONS_000391*2*2* in *trans*. Interestingly, the PC gene *XM_006*1*396*2*8*.*2* was regulated by lncRNA *TCONS_00002327* and *TCONS_000*1*9442* in *cis-* and *trans* simultaneously. Furthermore, the lncRNAs correlated with PC genes adopted multiple pathways, including one lncRNA correlated with one PC gene, one lncRNA correlated with several PC genes and several lncRNA correlated with one PC gene. *Dmrt1* (*XM_006137866*.*2*), *gata4* (*XM_014569886*.*1*) and *cyp19a* (*XM_006135075*.*2*) have been identified as key sex differentiation-related genes^2^ and were shown to be regulated by several lncRNAs actin in *cis* or in *trans* in the network. Therefore, the correlation network of lncRNAs and mRNAs indicated that lncRNAs act on PC genes both in *cis* and in *trans* in the process of sex differentiation in Chinese soft-shell turtles.

### Validation of differentially expressed lncRNAs and mRNAs

**Figure 6:**
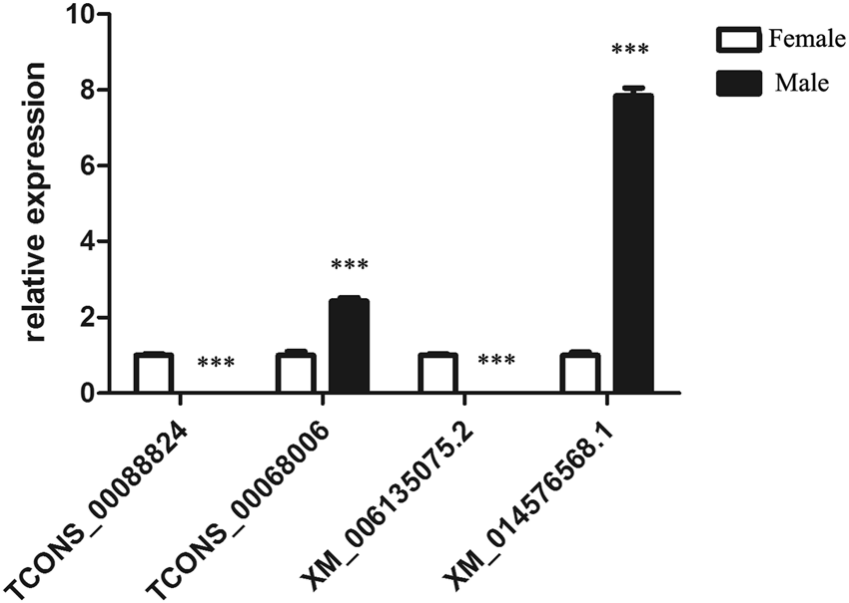
Validation of RNA-seq results by quantitative RT-PCR. Two lncRNAs and their target genes were analysed by quantitative RT-PCR. Data represent the mean ± 1 SD (n = 3). ^*^P < 0.05, ^**^P < 0.01, ^***^P <  0.001.

The differentially expressed lncRNA transcripts *TCONS_00088824* and *TCONS_00068006* and the PC genes *XM_006135075*.*2* (*Cyp19a*) and *XM_014576568*.*1* (*Sox9*) identified in the RNA-seq data were obtained according to gene expression, the lncRNA and mRNA regulatory network, and gene function to validate their expression patterns in female and male gonad tissues by qRT-PCR. The results confirmed consistent expression patterns of the two lncRNA transcripts and coding transcripts (Fig. 6), suggesting that the process used to identify putative lncRNAs was sufficiently stringent to correlate with most of the identified lncRNAs expressed *in vivo*. Interestingly, we identified a positive correlation between the expression of two lncRNA transcripts with target PC genes. The regulatory mechanism of these lncRNA-regulated targets requires further investigation.

## Discussion

LncRNAs have a disproportionately large impact on sex determination and sex differentiation processes that have been widely studied in humans and other mammals. Since the Chinese soft-shell turtle exhibits a sex-dependent dimorphic growth pattern^33^, the mechanisms underlying sex determination and sex differentiation in this species are of considerable interest. In this study, we performed genome wide analysis of lncRNAs from early-stage female and male gonads of Chinese soft-shell turtle to explore the sex differentiation-related candidate lncRNAs, thereby clarifying the sex differentiation processes in this species and also providing further elucidation of the functions of lncRNAs in this process in all reptiles.

In the present study, our systematic analysis of the RNA expression profiles in gonad tissues using RNA-seq revealed 5,994 putative lncRNA transcripts and 39,262 putative protein coding transcripts. In accordance with similar studies in different organisms, the identified lncRNA transcripts had fewer exons, shorter transcripts, and lower expressions than the identified protein coding transcripts^39^. We also found that the lncRNA transcripts in Chinese soft-shell turtle were longer than that the mean length of those in zebrafish (1,113 nt), goat (1,180 nt), human (1,000 nt) and mouse (550 nt)^40–42^, but shorter than those in chicken (2,941 nt)^43^. Furthermore, on average, lncRNA transcripts in Chinese soft-shell turtle contained fewer exons than those in zebrafish (2.8), goat (2.2), human (2.9) and mouse (3.7)^40–42^. Validation of two key deferentially expressed lncRNA transcripts by qRT-PCR yielded results were consistent those of the RNA-seq analysis, thus confirming the high quality of the identified lncRNAs.

LncRNAs do not encode proteins, which represents a major challenge in determining their function. Gene expression analyses are important in clarifying the potential function of these lncRNAs^44^. In this study, we screened out 932 specific-expression lncRNA transcripts in female gonad tissues and 449 specific-expression lncRNA transcripts in male gonad tissues (Fig. 2A). Furthermore, a large number of lncRNAs were found to be differentially expressed in female gonad tissues (Fig. 3A). Interestingly, we screened out 15 sex differentiation-related lncRNAs that were significantly different expressed in gonad tissues (Fig. 3C). These results indicated that lncRNAs may participate in the process of bipotential gonad differentiation into testis or ovary in Chinese soft-shell turtles.

LncRNAs have been reported to regulate PC gene expression either in *cis* or in *trans*^45^. *Cis-*acting lncRNAs act regulate the expression of a neighbouring gene on the same allele at the transcriptional or post-transcriptional level^46^. *Trans*-acting lncRNAs regulate the expression of genes that are located on other chromosomes^38^. Thus, in this study, we sought to identify potential regulatory targets of lncRNAs using the *cis* and *trans* model, which has been employed successfully to identify candidate regulatory targets of lncRNAs in animals and plants^47,48^. Using this strategy, we identified 933 *cis*-acting lncRNAs corresponding to 1,179 target and 5,990 *trans-*acting lncRNAs corresponding to 6,609 target genes. As previous shown in previous research, the functions of lncRNAs were mediated by their action on the PC genes. The *Xist* lncRNA interacts directly with SHARP to silence transcription of one X-chromosome during development in female mammals^28^. The lncRNA *Dmr* functions in *trans* splicing the *Dmrt1* transcript^29^. *Sxl* expression is regulated by a complex interaction network involving many lncRNAs^30^. In this study, we identified numerous lncRNAs associated with regulation of sex differentiation-related genes, including *dmrt1*, *sox9*, *cyp19a*, *sox3* and *sox8*, indicating that the initiation of sexual differentiation is regulated by the interaction between lncRNA and target genes. Most notably, we discovered a positive correlation between the expression of lncRNAs *TCONS_00088824* and *TCONS_00068006* and their respective target PC genes *XM_006135075*.*2* (*Cyp19a*) and *XM_014576568*.*1* (*Sox9*) (Fig. 6). This correlation may be the result of common regulation of local chromatin and further investigations are required to confirm the existence of authentic relationships between an lncRNA and its neighbouring targets in the regulation of sex differentiation in the Chinese soft-shell turtle.

LncRNA-mRNA correlation network analysis was performed to identify the most important sex differentiation-related genes as previously described^43^. Our analysis suggested potential lncRNAs candidates participate in sex differentiation through a complex lncRNA-mRNA regulation network (Fig. 5) by acting both in *cis* and in *trans* via multiple correlation pathways.

The findings of the present study provide evidence for the role of lncRNAs in sex differentiation in the Chinese soft-shell turtle. Furthermore, identification and annotation of the putative lncRNAs provides a basis for further clarification of the mechanisms underlying the regulation ofmRNA expression by lncRNA in sex determination in this species.

## Materials and Methods

### Animals and tissue preparation

Fertilised Chinese soft-shell turtle eggs were obtained from a fishery in Anhui Province, China. The eggs were placed with the animal pole upwards in sterilized and hatched at room temperature in our laboratory. At 30 days post-hatching, gonads were collected from turtles and immediately frozen in liquid nitrogen for storage at −80 °C prior to RNA extraction. All experiments in this research were performed according to the permit guidelines established by Anhui Agricultural University, and the experimental protocols were approved by the Animal Care and Use Committee of Anhui Agricultural University.

### RNA extraction, library construction, and sequencing

Total RNA was extracted using Trizol reagent (Invitrogen, USA) according to the manufacturer’s instructions. RNA purity and concentration were then evaluated using the NanoDrop 2000 (NanoDrop 2000/2000c, Thermo), RNA integrity was determined by 1% agarose gel electrophoresis and RNA quality was verified using the Agilent 2100 Bioanalyzer (Agilent Technologies, Santa Clara, CA, USA) as well as RNase free agarose gel electrophoresis^49–51^.

Following removal of rRNA using the Ribo-Zero™ Magnetic Gold Kit, the isolated mRNA was fragmented (200–500 nt) by adding fragmentation buffer. First-strand cDNA was then generated by reverse transcription using the isolated mRNA as a template and random hexamer primers. Second-strand cDNA was generated using RNase H and DNA polymerase I with specific dUTPs. After washing with EB buffer for the addition of poly(A) tails, the dsDNA fragments were ligated to the strand-marker adapters. The second-strand cDNA was degraded using uracil N-glycosylase. Following PCR and ligation of the sequencing adapters, the final cDNA library was constructed and sequenced in a single run on the Illumina platform (Illumina HiSeq™ 4000) using the paired-end technology. Base-calling and quality value calculations were performed by the Illumina HiSeq. 4000 to obtain 150 bp paired-end reads^52^.

### Transcriptome assembly and expression analysis

Clean data were obtained from the raw data by removing reads containing the following: adapters, >10% of poly(N), and low-quality (>50% of the bases had Phred quality scores ≤10). The Phred score (Q20) and GC content of the clean data were then calculated to identify the high quality clean data for all further analyses. The *Pelodiscus sinensis* reference genome and gene model annotation files were downloaded from the NCBI database (CHIR_1.0, NCBI). The reference genome was built using Bowtie v2.0.6 and paired-end clean reads were aligned using TopHat v2.0.14. The mapped reads from each library were assembled with Cufflinks v2.2.1 using default parameters, with the exception of ‘min-frags-per-transfrag = 0’ and ‘-library-type fr-firststrand’^53,54^.

Following assembly of the novel transcripts from the different libraries, putative lncRNAs were obtained by removal of the following: (1) Single exon transcripts and transcripts <200 bp; (2) The remaining transcripts that overlapped (>1 bp) with *P*. *sinensis* protein coding genes; (3) Transcripts likely to be assembly artefacts or PCR run-on fragments according to the class code annotated by cuff-compare. Transcripts with cuff-compare classcodes “u, i, j, x, c, e, o” were defined as novel transcripts. (4) Transcripts with Coding Potential Calculator (CPC) scores >0 and Coding-Non-Coding-Index (CNCI) scores >0. The details of this process are shown in Fig. 1.

For each library, the FPKM scores for the lncRNAs and coding genes were calculated using RSEM and differentially expressed lncRNAs between any two libraries were identified by edgeR (release 3.2). The thresholds used to evaluate the statistical significance of differences in lncRNA expression were defined as FDR <0.05 and an absolute value of the log_2_ (fold change) >1. The clusters obtained in this systematic analysis of differentially expressed lncRNAs in the four libraries were analysed using the Heatmaps software package in R^55,56^.

### Target gene prediction and functional enrichment analysis

*Cis*-acting lncRNAs function by targeting neighbouring genes^57,58^. In this study, we searched for coding genes in the regions located 10-kb upstream and downstream of all the identified lncRNAs for prediction of their functional roles. The RNAplex software (http://www.tbi.univie.ac.at/RNA/RNAplex.1.html) was used to predict the complementary correlation of antisense lncRNA and mRNA to reveal their interactions. This program contains the ViennaRNA package, which predicts the best base pairing through calculation of the minimum free energy of the thermodynamic structure. LncRNAs also mediate *trans*-regulation of distant genes. LncRNAs and coding genes were identified as those with Pearson’s correlation coefficients ≥ ± 0.9.

KEGG pathway analysis according to KEGG (http://www.genome.jp/kegg/) pathway analysis was performed to investigate the roles of all differentially expressed mRNAs^40,59^.

### Validation of differentially expressed lncRNAs and mRNAs by quantitative RT-PCR

Two differentially expressed lncRNAs and two target genes were selected to validate the RNA-seq data by qRT-PCR using previously described methods^60^. Primers were designed using Primer3 (sequences are shown in Table 2) and evaluated using BLAST at NCBI. *GAPDH* was used as the internal control. Each sample was analysed in three independent experiments.

**Table 2:** List of primers used in the qRT-PCR validation of RNA-seq.

Gene ID	Forward primer	Reverse primer
*XM_014576568*.*1*	TTCCGACCGCTAAAACGA	CACCGTTTAGCCATTGTTGTT
*TCONS_00068006*	CCCACCCCCTTTTTGACT	AAAGTTTTGCCTGGTCGCT
*XM_006135075*.*2*	GGAAGCAAACTTGGATTACAG	ATGTTTTCCAGTCCAGCAGTA
*TCONS_00088824*	GTATCCATTAGGTAACGCTGAC	AAATCCCACTGGAAGTCAATAG
*GAPDH*	CCGTGTTCCAACTCCCAATG	GGCAGCCTTCATCACCTTCTT

### Construction of lncRNA and target gene network

Correlation analysis of the differentially expressed lncRNAs and mRNAs was performed to identify interactions. This information was then used to construct a co-expression network using Cytoscape software (The Cytoscape Consortium, San Diego, CA, USA).

### Statistical analyses

Statistical analyses were performed using SAS software version 9.0 (SAS, Cary, North Carolina, USA). All data were analysed using one-way analysis of variance (ANOVA). Homogeneity of variances was evaluated using Levene’s test followed by Student’s *t*-test. *P < *0.05 was considered to indicate statistical significance.

### Accession numbers

The sequencing data were submitted to the Sequence Read Archive in the NCBI (accession No.: SRP125617).

## Electronic supplementary material

Supplementary information

Dataset 1

Dataset 2

Dataset 3

Dataset 4

Dataset 5

Dataset 6

Dataset 7
