# Supplementary material for: Screening and characterisation of sex differentiation-related long non-coding RNAs in Chinese soft-shell turtle (Pelodiscus sinensis)
Source: Sci Rep. 2018 Jun 5;8:8630. doi: 10.1038/s41598-018-26841-3 (PMC5988831; doi:10.1038/s41598-018-26841-3)
Supplement: Supplementary file 1 — Supplementary information [file 41598_2018_26841_MOESM1_ESM.pdf]

Screening and characterisation of sex differentiation-related long non-coding RNAs in Chinese soft-shell turtle (*Pelodiscus sinensis*)

Jun Zhang[a, 1], Peng Yu [a, b, c, 1], Qinyan Zhou[a], Xilei Li[a], Shuquan Ding[a], Shiping Su[a], Xiaohua Zhang[a], Xiaoli Yang[a], Weishang Zhou[a], Quan Wan[a, *], Jian-Fang Gui[b, c, *]

Supplementary information

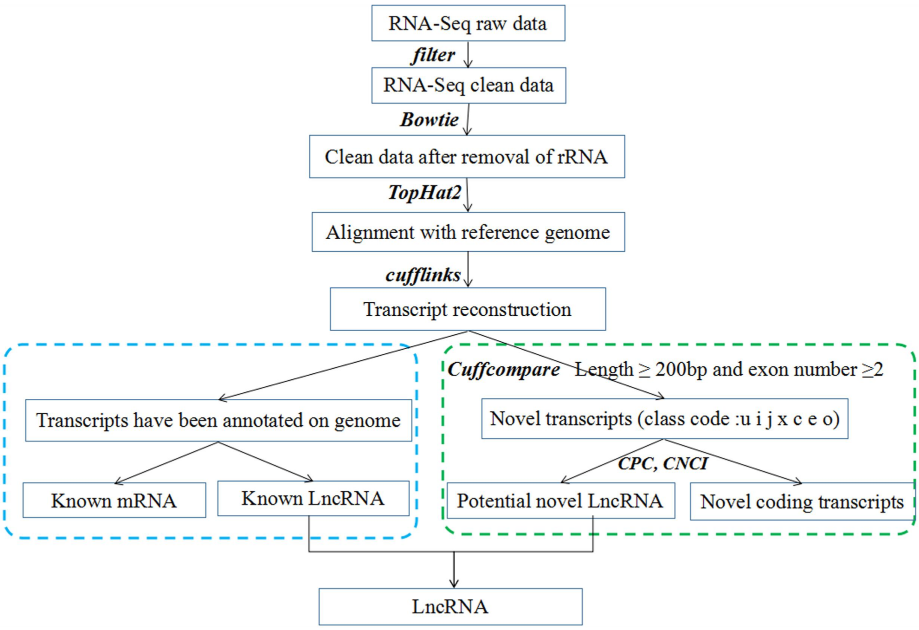

Figure S1 Identification pipeline for lncRNAs and protein coding transcripts. Each step is described in detail in the methods section.

Supplementary File S1. Reads produced by the Illumina HiSeq 4000 platform.

Supplementary File S2. The FPKM of differentially expressed transcripts.

Supplementary File S3. Dysregulated sex differentiation-related lncRNAs.

Supplementary File S4. Dysregulated sex differentiation-related mRNAs.

Supplementary File S5. The protein coding genes as potential targets in the regions located 10-kb upstream and downstream of the lncRNAs.

Supplementary File S6. Potential protein coding target genes of the *trans*-acting lncRNAs.

Supplementary File S7. KEGG pathway analysis of predicted targets of *cis*-acting lncRNAs.
